# Inhibiting cytoplasmic accumulation of HuR synergizes genotoxic agents in urothelial carcinoma of the bladder

**DOI:** 10.18632/oncotarget.9932

**Published:** 2016-06-09

**Authors:** Jiawei Guo, Jing Lv, Siyu Chang, Zhi Chen, Weiqiang Lu, Chuanliang Xu, Mingyao Liu, Xiufeng Pang

**Affiliations:** ^1^ Shanghai Key Laboratory of Regulatory Biology, Institute of Biomedical Sciences and School of Life Sciences, East China Normal University, Shanghai 200241, China; ^2^ Department of Urology, Shanghai Changhai Hospital, Second Military Medical University, Shanghai 200433, China; ^3^ Institute of Biosciences and Technology, Department of Molecular and Cellular Medicine, Texas A&M University Health Science Center, Houston, Texas 77030, USA

**Keywords:** RNA-binding protein, pyrvinium pamoate, DNA damage response, chemosensitivity, urothelial carcinoma of the bladder

## Abstract

HuR, an RNA-binding protein, post-transcriptionally regulates nearly 4% of encoding proteins implicated in cell survival. Here we show that HuR is required for the efficacy of chemotherapies in urothelial carcinoma of the bladder. We identify pyrvinium pamoate, an FDA-approved anthelminthic drug, as a novel HuR inhibitor that dose-dependently inhibited cytoplasmic accumulation of HuR. Combining pyrvinium pamoate with chemotherapeutic agents (e.g. cisplatin, doxorubicin, vincristine and oxaliplatin) not only led to enhanced cytotoxicity in bladder cancer cells but also synergistically suppressed the growth of patient-derived bladder tumor xenografts in mice (*P* < 0.001). Mechanistically, pyrvinium pamoate promoted nuclear import of HuR by activating the AMP-activated kinase/importin α1 cascade and blocked HuR nucleo-cytoplasmic translocation by inhibiting the checkpoint kinase1/cyclin-dependent kinase 1 pathway. Notably, pyrvinium pamoate-additive treatment increased DNA double-strand breaks as indicated by elevated γH2AX expression, suggesting an involvement of DNA damage response. We further found that pyrvinium pamoate dramatically downregulated several key DNA repair genes in genotoxically-stressed cells, including DNA ligase IV and BRCA2, leading to unbearable genomic instability and cell death. Collectively, our findings are the first to characterize a clinical HuR inhibitor and provide a novel therapeutically tractable strategy by targeting cytoplasmic translocation of HuR for treatment of urothelial carcinoma of the bladder.

## INTRODUCTION

Despite achievements in understanding the molecular pathogenesis of urothelial carcinoma of the bladder (UCB), no target-selective agents have been approved either as monotherapy or in combination with traditional chemotherapy in the treatment of metastatic or recurrent UCB [1]. Systemic chemotherapy with a platinum-based regimen remains the current standard of care for UCB patients [2, 3]. Unfortunately, approximately half of UCB patients are not eligible for cisplatin-based treatment regimens due to poor response [4] or unbearable renal failure [5]. Therefore, enhancing the efficacy of current chemotherapies under a tolerable dosage is of paramount importance. Since nearly all the chemotherapeutic agents used in UCB treatment are genotoxic agents, clinical improvements will likely come from strategies aimed at augmenting genotoxic agents-mediated DNA damage.

The most lethal form of DNA damage caused by genotoxic agents is DNA double-strand breaks (DSBs). DSBs trigger a series of sophisticated DNA repair mechanisms, called DNA damage response (DDR), to neutralize DNA damage [6]. DDR includes cell cycle arrest and DNA repair events where homologous recombination (HR) and non-homologous end joining (NHEJ) help cells to maintain genomic stability and escape from chemotherapy [7, 8]. Therefore, interfering with the DDR process is thought to be an intriguing way to potentiate chemotherapies [6]. However, despite achievements in the development of PARP inhibitors [9] as well as Chk1 inhibitors [10], unexpected problems occurred as complete inhibition of HR or NHEJ may be lethal to all dividing cells [6]. To minimize unwanted toxicity on normal tissues, temporarily interfering with the DDR pathway is likely to be more feasible. Notably, subcellular distribution of RNA-binding proteins (RBPs) is quite responsive to DNA damage [11, 12]. RBPs binding to specific pre-mRNAs and mRNAs selectively regulate DDR genes at multiple post-transcriptional levels (e.g. translation rate and mRNA stability) and finally lead genome stability [12].

HuR, one of the best-studied RBPs implicated in DDR, is indispensable for the stabilization of short-lived mRNAs [13]. The 326 amino acid-long HuR recognizes AU-rich RNA motifs in the 3′-UTR (3′-untranslated region) and binds to its target mRNAs through its RNA recognition motifs [14]. As a sensor protein, HuR's activation is a complicated process including subcellular translocation, phosphorylation as well as homodimer formation [11, 15, 16]. Although HuR is predominantly located in the nucleus, its nucleo-cytoplasmic translocation during DNA damage response has been implicated in genomic stability maintenance as well as cell fate decision [17, 18]. HuR-mediated DNA damage response is partially attributed to its ability to stabilize several mRNAs of key DDR-related genes, including p53 [19], p21 [20], RhoB [21] and WEE1 [22]. HuR inhibition has been described to be antineoplastic or antiangiogenic, demonstrating the importance of HuR as a potential target in cancer therapy [15]. In addition, the correlation between HuR cytoplasmic accumulation and poor prognosis of bladder cancer patients has recently been discovered [23, 24]. Since HuR activation may help the DNA repair process during DDR, blocking HuR-mediated responses is expected to improve the efficacy of genotoxic agents. Under this hypothesis, we find that inhibiting HuR is required for chemotherapeutic efficacy in UCB and further identified pyrvinium pamoate as a novel inhibitor of HuR cytoplasmic translocation. Pyrvinium pamoate potentiates chemotherapeutic agents in several preclinical cancer models *in vitro* and *in vivo*. Furthermore, a dual mechanism involving activation of the AMP-activated protein kinase (AMPK)/importin α1 cascade and inhibition of the Chk1/Cdk1 pathway contribute to pyrvinium pamoate-mediated suppression of HuR cytoplasmic translocation. As a consequence of HuR inhibition, several DNA repair factors decrease their expression due to loss of mRNA stability. Collectively, our results establish a paradigm whereby systemic modulation of cytoplasmic abundance of HuR is sufficient to sensitize chemotherapy and a mechanism of HuR in DNA damage response.

## RESULTS

### Identification of pyrvinium pamoate as a novel HuR inhibitor

It has been reported that HuR functionally translocates into the cytoplasm under DNA-damaging stress conditions in pancreatic cancer cells [22]. To confirm whether HuR translocation happens in urothelial carcinoma of the bladder, we first treated bladder cancer cells with four first-line chemotherapeutic agents, including cisplatin, oxaliplatin, doxorubicin and vincristine. The results showed that HuR was highly abundant in the cytoplasm of all treated cells, whereas total HuR protein level in whole cell lysates exhibited no significant change (Supplementary Figure S1A). Such effect was a time-dependent action as indicated by time-course treatments of doxorubicin (Supplementary Figure S1B). Results from immunofluorescence assays consistently showed that HuR was highly responsive to chemotherapeutic stress by translocating from the nucleus to the cytoplasm (Supplementary Figure S1C).

**Figure 1:**
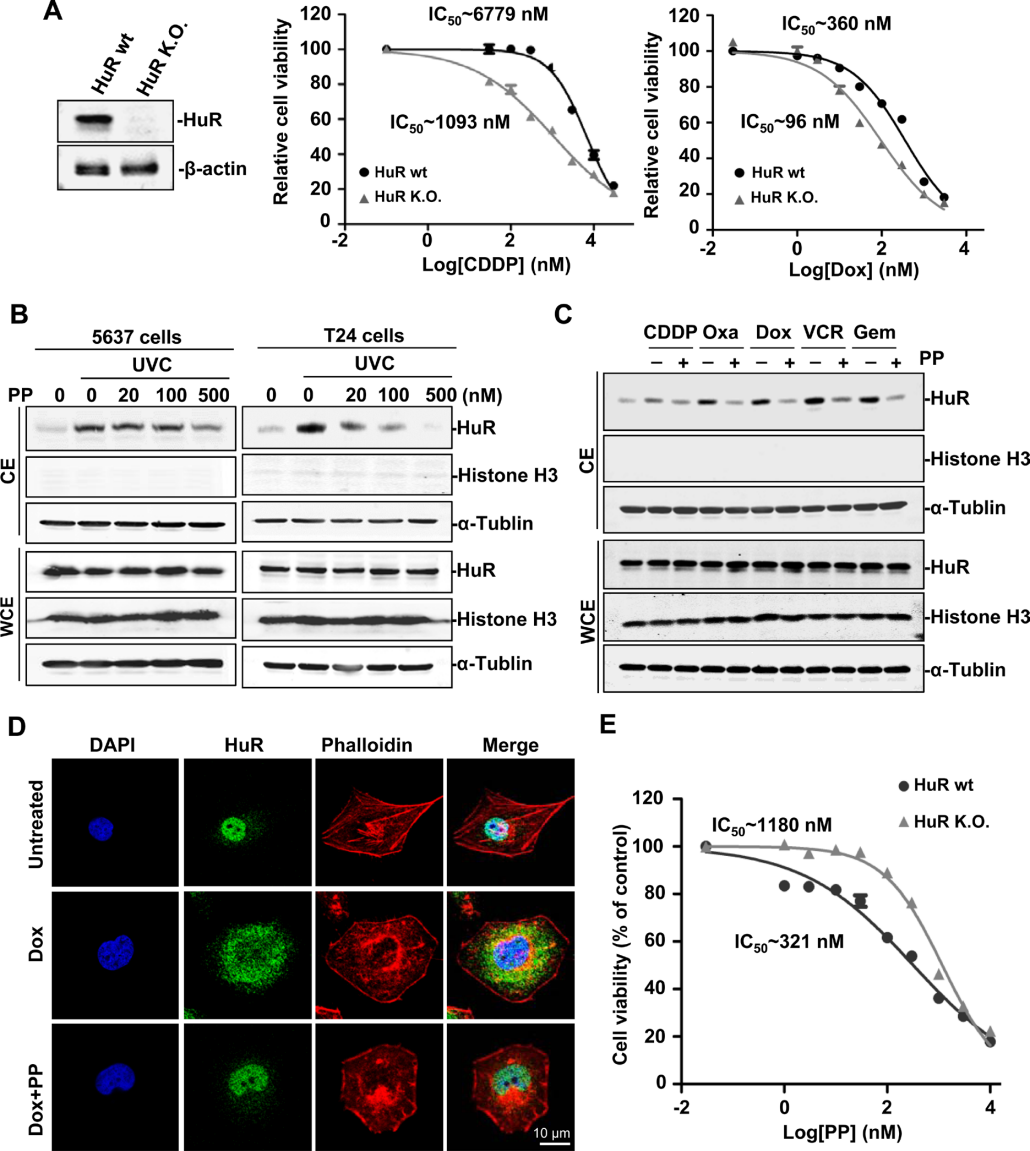
Identification of pyrvinium pamoate as an effective inhibitor of HuR (**A**) HuR ablation potentiates the efficacy of genotoxic agents. Left, HuR isogenic cell lines; right, HuR deletion potentiated chemotherapeutic efficacy in 5637 cancer cells. (**B**) Pyrvinium pamoate inhibits the UVC-triggered increase of HuR cytoplasmic accumulation in a concentration-dependent manner. (**C**) Pyrvinium pamoate blocks genotoxic agent-triggered cytoplasmic translocation of HuR. 5637 cells were treated with different genotoxic agents alone or in combination with 100 nmol/L of pyrvinium pamoate for 48 h. (**D**) Pyrvinium pamoate blocks doxorubicin-triggered cytoplasmic accumulation of HuR by immunofluorescence. (**E**) HuR-null cells are more resistant to pyrvinium pamoate's treatment compared to their wild-type counterparts. IC_50_s were calculated by GraphPad Prism Software. *Dot*, mean; *bars*, standard deviation. CE, cytoplasmic extracts; WCE, whole-cell extracts; PP, pyrvinium pamoate; CDDP, cisplatin; Oxa, oxaliplatin; Dox, doxorubicin; VCR, vincristine; Gem, gemcitabine; wt, wild-type; K.O., knockout.

We tested if HuR played a role in chemotherapeutic efficacy. We generated a pair of isogenic cell lines by totally deleting HuR in 5637 cells. As shown in Figure 1A, HuR-null cells were more vulnerable to the proliferative inhibition caused by cisplatin or doxorubicin. The IC_50_s of cisplatin and doxorubicin decreased by nearly 83% and 75% in HuR-null cells, respectively. These data indicated that HuR activity reduced the efficacy of genotoxic agents and targeting HuR cytoplasmic translocation may enhance the sensitivity of UCB cells to current therapy.

No clinically applicable HuR inhibitors have been characterized until now. Therefore, we conducted a primary screen for such agents from a library of FDA-approved drugs using a high-throughput screen system by inserting an HuR-recognized AU-rich sequence into the 3′-UTR of firefly luciferase (FLuc) mRNA (Supplementary Figure S2A). In order to efficiently activate HuR by genotoxic stress, we exposed transfected cells to UVC radiation in the primary screen. HuR activated by UVC was able to bind to the AU-rich elements downstream of FLuc and thus stabilized its mRNAs. The expression of FLuc was relatively increased under UVC exposure compared to renilla luciferase (RLuc) who lacked ARE in its 3′-UTR. Agents that interfered with HuR's activation were expected to decrease the ratio of FLuc luminescence compared to RLuc. A novel finding was that pyrvinium pamoate showed the most effective action and significantly inhibited the UVC-triggered increase of the FLuc/RLuc ratio (Supplementary Figure S2B). To confirm the inhibitory effect of pyrvinium pamoate on HuR, we next examined the expression of *p53*, *p21* and *PTMA*, which are UVC-responsive mRNAs with known HuR recognized AREs in their 3′-UTR. The results showed that pyrvinium pamoate dose-dependently inhibited their expression (Supplementary Figure S3).

Since translocation from the nucleus to the cytoplasm acts as the key functional regulatory step for HuR activation [14], especially in stressed cells [11], we further examined the effects of pyrvinium pamoate on HuR subcellular distribution. As shown in Figure 1B, pyrvinium pamoate blocked UVC-mediated cytoplasmic accumulation of HuR in a concentration-dependent manner. We next investigate whether pyrvinium pamoate was also effective in chemotherapy-induced HuR activation. The results showed that pyrvinium pamoate potently suppressed nucleo-cytoplasmic translocation of HuR in chemotherapy-stressed conditions (Figure 1C). Similarity was observed by immunofluorescence assays (Figure 1D). Together, these data indicated that pyrvinium pamoate was an effective inhibitor of HuR cytoplasmic translocation.

Given the potent effect of pyrvinium pamoate on HuR translocation, we verified whether HuR primarily contributed to the action of pyrvinium pamoate by using HuR isogenic cell lines. The result showed the HuR-null cells were nearly three-fold more resistant to pyrvinium pamoate treatment compared to their wild-type counterparts (Figure 1E), suggesting that HuR was a likely potential target of pyrvinium pamoate.

### Pyrvinium pamoate potentiates genotoxic agents

**Figure 2:**
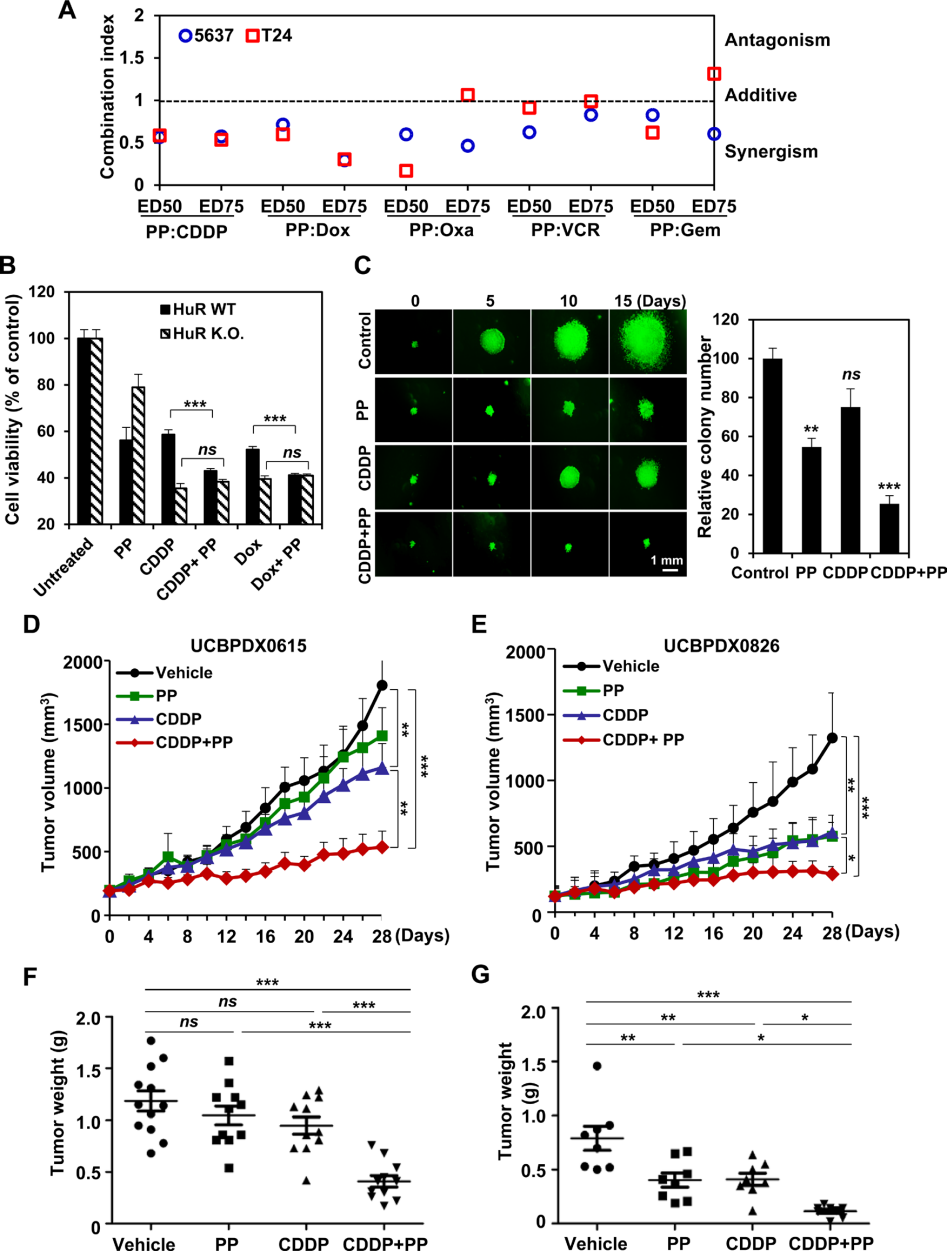
Pyrvinium pamoate potentiates genotoxic agents *in vitro* and *in vivo* (**A**) The synergy of pyrvinium pamoate with five different genotoxic agents. Combinatorial index values at 50% effective dose (ED50) or 75% effective dose (ED75) were calculated using CalcuSyn software. (**B**) HuR is required for the synergy of pyrvinium pamoate with chemotherapeutic agents. Cell viability was determined, and a two-tailed unpaired *t*-test was performed. (**C**) Addition of pyrvinium pamoate enhances chemotherapeutic efficacy in spheroid growth assays. 5637 cells were exposed to 320 nmol/L of pyrvinium pamoate, 6.8 μmol/L of cisplatin or the combined treatment at their half concentrations for 15 days. Left, representative spheroids; right, colony number. (**D** and **E**) Tumor volume of two primary bladder cancer xenograft models. Tumor volumes were recorded every other day during treatments (*n* = 8–12 each group). (**F** and **G**) Tumor weight in mice. Solid tumor weight was measured on day 28 (*n* = 8–12 each group). Statistical comparisons were performed by One-way ANOVA analysis. *Columns* and *dots,* mean; *bars*, standard deviation. *ns*, not significant; *P < 0.05; **P < 0.01; ***P < 0.001. PP, pyrvinium pamoate; CDDP, cisplatin; Dox, doxorubicin; wt, wild-type; K.O., knockout.

Given that HuR played a critical role in chemotherapeutic efficacy and served as a potential target of pyrvinium pamoate, we sought to test if pyrvinium pamoate was capable of sensitizing cells to genotoxic agents. The combinational indices (CIs) at 50% effective dose or 75% effective dose of agents were calculated to determine drug interactions. The results showed that pyrvinium pamoate and genotoxic agents (doxorubicin, cisplatin, oxaliplatin, vincristine and gemcitabine) were synergistic in both 5637 and T24 cells (Figure 2A).

When treated the isogenic cells with different drug combinations, we found that pyrvinium pamoate lost potency to sensitize chemotherapy (Figure 2B), suggesting that the synergistic efficacy of pyrvinium pamoate and chemotherapeutic agents was primarily dependent on HuR. A 3-D colony formation assay further showed that a significant improvement of cisplatin efficacy was observed once pyrvinium pamoate was added (*P* < 0.001), although the concentrations of pyrvinium pamoate and cisplatin were cut by half (Figure 2C, left). The combined treatment decreased both colony size and colony number in a significant manner compared to either pyrvinium pamoate or cisplatin alone (Figure 2C, right).

We next confirmed the synergy *in vivo*. Two primary bladder tumor xenograft mouse models (UCBPDX0615 and UCBPDX0826) were set up. Compared to single agent alone, the combined regimen produced a more significant decrease in tumor volume and tumor weight (Figure 2D–2G). Notably, the efficacy of the combination was durable since the average tumor volume was almost comparable to the baseline during 4-week treatment (Figure 2D, 2E). Besides, the combination treatment was quite tolerable as mouse body weights were not obviously affected (Supplementary Figure S4). Our *in vitro* and *in vivo* data indicated the feasibility of augmenting chemotherapeutic efficacy with a pyrvinium pamoate-combination strategy.

### Pyrvinium pamoate promotes nuclear import of HuR by activating the AMPK/importin α1 signaling cascade

**Figure 3:**
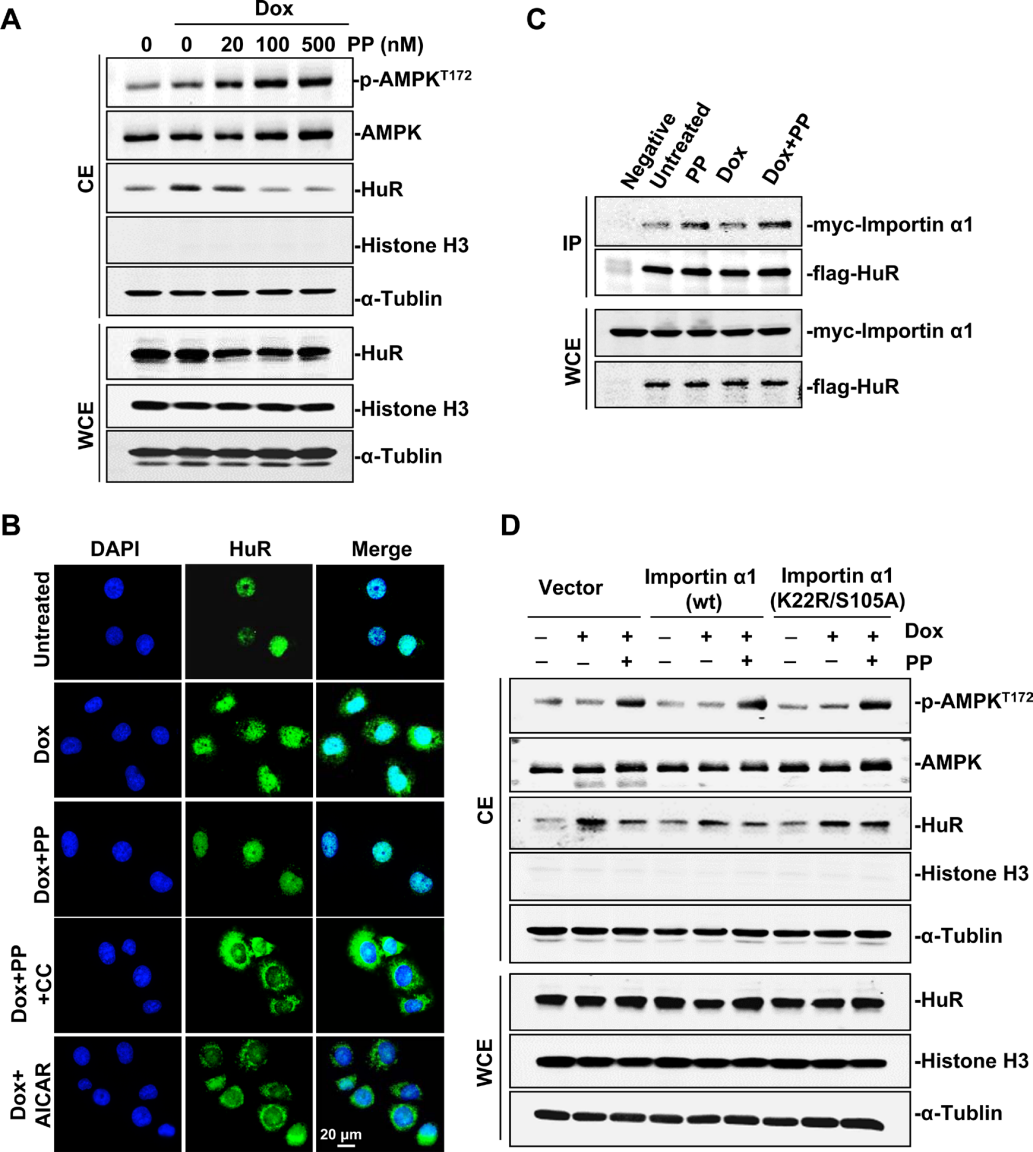
Pyrvinium pamoate activates the AMPK/importin α1 cascade (**A**) Pyrvinium pamoate activates AMPK and decreases HuR cytoplasmic abundance in a dose-dependent manner. (**B**) Immunofluorescence assays shows that pyrvinium pamoate inhibits doxorubicin-triggered cytoplasmic translocation of HuR by activating the AMPK pathway. 5637 cells were treated with doxorubicin (360 nmol/L) for 12 h, followed by indicated treatments (100 nmol/L pyrvinium pamoate, 10 μmol/L compound C and 2 mmol/L AICAR) for an additional 6 h. Immunofluorescence staining for HuR was performed (magnification, 40×). (**C**) Pyrvinium pamoate improves the interaction of HuR and importin α1. Cells transfected with equivalent amount of flag-pcDNA3.1 and myc-importin α1 served as the negative control. (**D**) Importin α1 is required for pyrvinium pamoate-mediated HuR nuclear import. 5637 cells were transfected with pcDNA3.1 or importin α1 (wild-type) or importin α1 (K22R/S105A, dual-site mutation). CE, cytoplasmic extracts; WCE, whole-cell extracts; Dox, doxorubicin; PP, pyrvinium pamoate; CC, compound C; AICAR, sodium azide, 5-amino-imidazole-4-carboxamide riboside.

The above findings prompted a further exploration of molecular basis underlying how pyrvinium pamoate-mediated the decrease of HuR cytoplasmic accumulation. Pyrvinium pamoate has been reported to suppress mitochondrial energy metabolism by inhibiting the NADH-fumarate reductase system [25]. Our results confirmed that treatment of pyrvinium pamoate led to a rapid time-dependent decrease of the ATP level in bladder cancer cells (Supplementary Figure S5). Given that AMP-activated protein kinase (AMPK) activation was rather responsive to decreased ATP and highly involved in the regulation of HuR, we investigated the effect of pyrvinium pamoate on AMPK signaling. We found that pyrvinium pamoate dose-dependently activated AMPK, coupling with a decrease of cytoplasmic HuR (Figure 3A), suggesting a potential role for AMPK in pyrvinium pamoate regulation of HuR. Rescue assays by immunofluorescence showed that AICAR, an AMPK activator, exhibited similar action as pyrvinium pamoate, whereas compound C, an AMPK inhibitor, squeezed HuR out of the nuclei even in the presence of pyrvinium pamoate (Figure 3B), indicating a pivotal role of AMPK in pyrvinium pamoate-mediated inhibition of HuR cytoplasmic accumulation.

Importin α1 is the only characterized transporter known to conduct nuclear import of HuR downstream of AMPK [26]. Our results showed that pyrvinium pamoate improved the binding of importin α1 and HuR under both doxorubicin-stressed and control conditions (Figure 3C), implying a potential involvement of importin α1 in pyrvinium pamoate-mediated HuR nuclear import. Since AMPK activates importin α1 by promoting acetylation at K22 as well as phosphorylation at S105 [26], we examined whether constitutive expression of importin 1 mutated at these sites (K22R/S105A dual mutant) would block the effect of pyrvinium pamoate. Our results showed that constitutive overexpression of importin α1 in 5637 cells weakened doxorubicin-induced HuR cytoplasmic accumulation, and the treatment of pyrvinium pamoate produced a further decrease (Figure 3D). In contrast, HuR in 5637 cells with the K22R/S105A mutant variant failed to respond to pyrvinium pamoate regardless of the activated AMPK status (Figure 3D). These data suggested that pyrvinium pamoate promoted HuR nuclear import and thus decreased HuR cytoplasmic accumulation through activating the AMPK/importin α1 cascade.

### Pyrvinium pamoate suppresses HuR nucleo-cytoplasmic translocation by inhibiting the Chk1/Cdk1 signaling pathway

**Figure 4:**
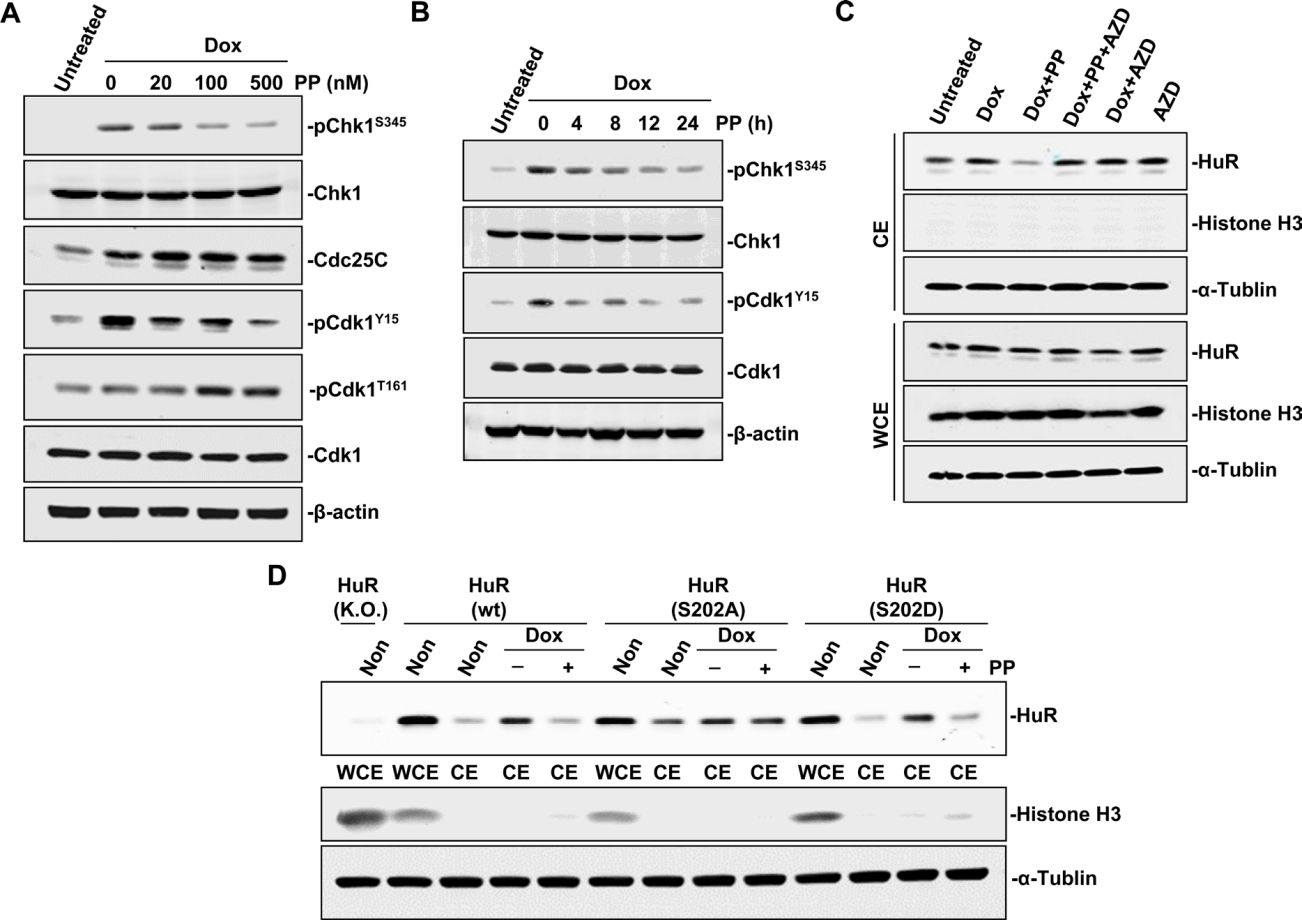
Pyrvinium pamoate interferes with the Chk1/Cdk1 pathway (**A** and **B**) Pyrvinium pamoate suppresses Chk1 and activated Cdk1 in dose-dependent (A) and time-dependent (B) manners. (**C**) Cdk1 inhibition rescues pyrvinium pamoate-mediated HuR cytoplasmic translocation. 5637 cells were treated with doxorubicin (360 nmol/L) for 12 h, followed by treatments with pyrvinium pamoate (100 nmol/L) and/or AZD5438 (10 μmol/L) for an additional 6 h, respectively. (**D**) HuR mutation at S202 site affects pyrvinium pamoate-mediated inhibition of HuR cytoplasmic translocation. Non, non-treatment; Dox, doxorubicin; PP, pyrvinium pamoate; AZD, AZD5438.

Apart from transporter interaction, phosphorylation of specific HuR amino acids affects its subcellular translocation as well [11]. Since checkpoint kinase 1 (Chk1) plays a vital role in genotoxic stress [27], and its downstream effector, cyclin-dependent kinase 1 (Cdk1), is implicated in HuR phosphorylation at S202 (pS202) [28], we investigated whether pyrvinium pamoate treatment affected the Chk1/Cdk1 signaling cascade. The results showed that doxorubicin activated Chk1 at S345, whereas pyrvinium pamoate inhibited this process in a time- and dose-dependent manner (Figure 4A and 4B). The weakened phosphorylation of Chk1 at S345 raises Cdk1 activity by two independent phosphorylation events: (*i*) augmented Cdk7-mediated phosphorylation of Cdk1 at T161, and (*ii*) attenuated Y15 phosphorylation of Cdk1 (an inhibitory phosphorylation site) by increasing phosphatase cdc25C level [29]. We found that both events contributed to pyrvinium pamoate-mediated the activation of Cdk1 under doxorubicin-stressed condition, where pyrvinium pamoate dose-dependently increased Cdk1 phosphorylation at T161 and the expression of cdc25C, and decreased pY15 Cdk1 phosphorylation (Figure 4A). This evidence supports the assumption that pyrvinium pamoate interfered with doxorubicin triggering the activation of the Chk1/Cdk1 signaling pathway. We next carried out a rescue assay using a Cdk1 selective inhibitor, AZD5438. As shown in Figure 4C, pyrvinium pamoate no longer suppressed doxorubicin-triggered HuR cytoplasmic accumulation in the presence of AZD5438.

HuR phosphorylation at S202 is the only known mechanism in Cdk1-regulated HuR translocation [28]. We found that baseline cytoplasmic HuR was more abundant in cells expressing the S202A HuR mutant than in cells with the S202D HuR mutant (Figure 4D). Cells harboring S202D HuR and HuR wild-type plasmids exhibited a similar response to treatment with pyrvinium pamoate, whereas HuR failed to translocate in the presence of pyrvinium pamoate in cells with non-phosphorylatable variant HuR (S202A), suggesting that pyrvinium pamoate suppressed HuR translocation from the nucleus to the cytoplasm by promoting a Cdk1-dependent phosphorylation of HuR at S202.

### Pyrvinium pamoate augments chemotherapy-mediated DNA double-strand breaks

**Figure 5:**
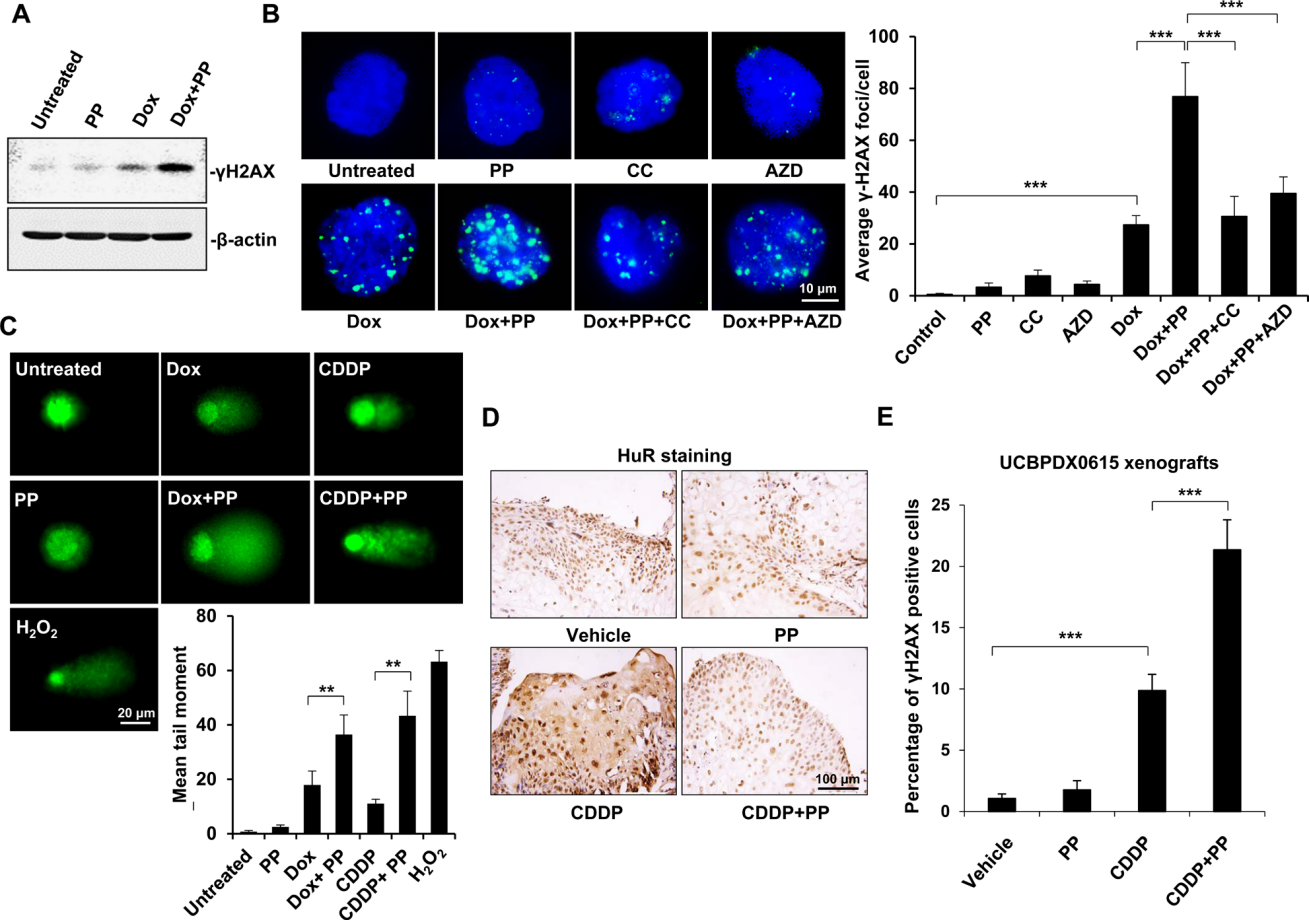
Pyrvinium pamoate enhances DNA double-strand breaks *in vitro* and *in vivo* (**A**) Pyrvinium pamoate enhances doxorubicin-induced DNA double-strand breaks in 5637 cells as indicated by increased γH2AX levels. (**B**) AMPK or Cdk1 inhibition weakens pyrvinium pamoate-mediated increase of γH2AX levels during doxorubicin's treatment. Immunofluorescence assay were conducted for γH2AX staining (magnification, 100×). Left, representative images; right, quantification of γH2AX foci. (**C**) Pyrvinium pamoate strengthens DNA double-strand breaks in an alkaline comet assay. H_2_O_2_ at concentration of 200 μmol/L was used as a positive control. Average tail moments were measured from 50 comet tails of each group. *Columns*, mean; *bars*, standard deviation. (**D**) Pyrvinium pamoate reverses cisplatin-triggered HuR cytoplasmic accumulation in tumors from the primary bladder tumor xenograft mouse model (UCBPDX0615). Representative images were shown (magnification, 20×). (**E**) Addition of pyrvinium pamoate leads to a significantly higher level of γH2AX in tumor tissues. Percentage of γH2AX positive cells in tumors from xenograft mouse model (UCBPDX0615) was shown. *Columns*, mean; *error bars*, standard deviation. *P < 0.05; **P < 0.01; ***P < 0.001. CDDP, cisplatin; Dox, doxorubicin; PP, pyrvinium pamoate; CC, compound C; AZD, AZD5438.

Given that DNA double-strand breaks (DSBs) play a vital role in the toxicity of genotoxic agents, we next examined whether pyrvinium pamoate enhanced genotoxic agent-triggered DNA damage. As a specific marker of DSBs, γH2AX reflects the content of unrepaired DSBs within cells [30]. Doxorubicin treatment alone triggered phosphorylation of γH2AX, whereas a much higher level of γH2AX was observed after pyrvinium pamoate was added (Figure 5A), suggesting increased DNA damage. We found that doxorubicin alone markedly triggered DSBs, whereas pyrvinium pamoate potentiated DSBs to a greater extent. Intriguingly, pyrvinium pamoate did not induce DSBs as a single agent (Figure 5B), suggesting the feasibility of pyrvinium pamoate in such a combinatorial regimen. Furthermore, compound C as well as AZD5438 relieved pyrvinium pamoate-triggered DSBs (Figure 5B), which additionally demonstrated the dual mechanism involved in pyrvinium pamoate's action on HuR.

An alkaline comet assay showed that both doxorubicin and cisplatin yielded a tail moment; however, a much longer tail was observed when either of them was in combination with pyrvinium pamoate (Figure 5C). It was notable that DSBs enhanced by pyrvinium pamoate did not result from additive DNA damage, as pyrvinium pamoate alone seemed insufficient to trigger tail moment lengthening (Figure 5C).

We also conducted immunohistochemistry on UCBPDX0615 xenografts, and the results showed that pyrvinium pamoate inhibited cisplatin-mediated cytoplasmic accumulation of HuR in a durable manner (Figure 5D). Meanwhile, addition of pyrvinium pamoate markedly increased cisplatin-triggered DSBs, as indicated by significantly increased percentage of γH2AX-positive tumor cells (Figure 5E).

### Pyrvinium pamoate suppresses HuR-mediated stabilization of DNA repair genes

**Figure 6:**
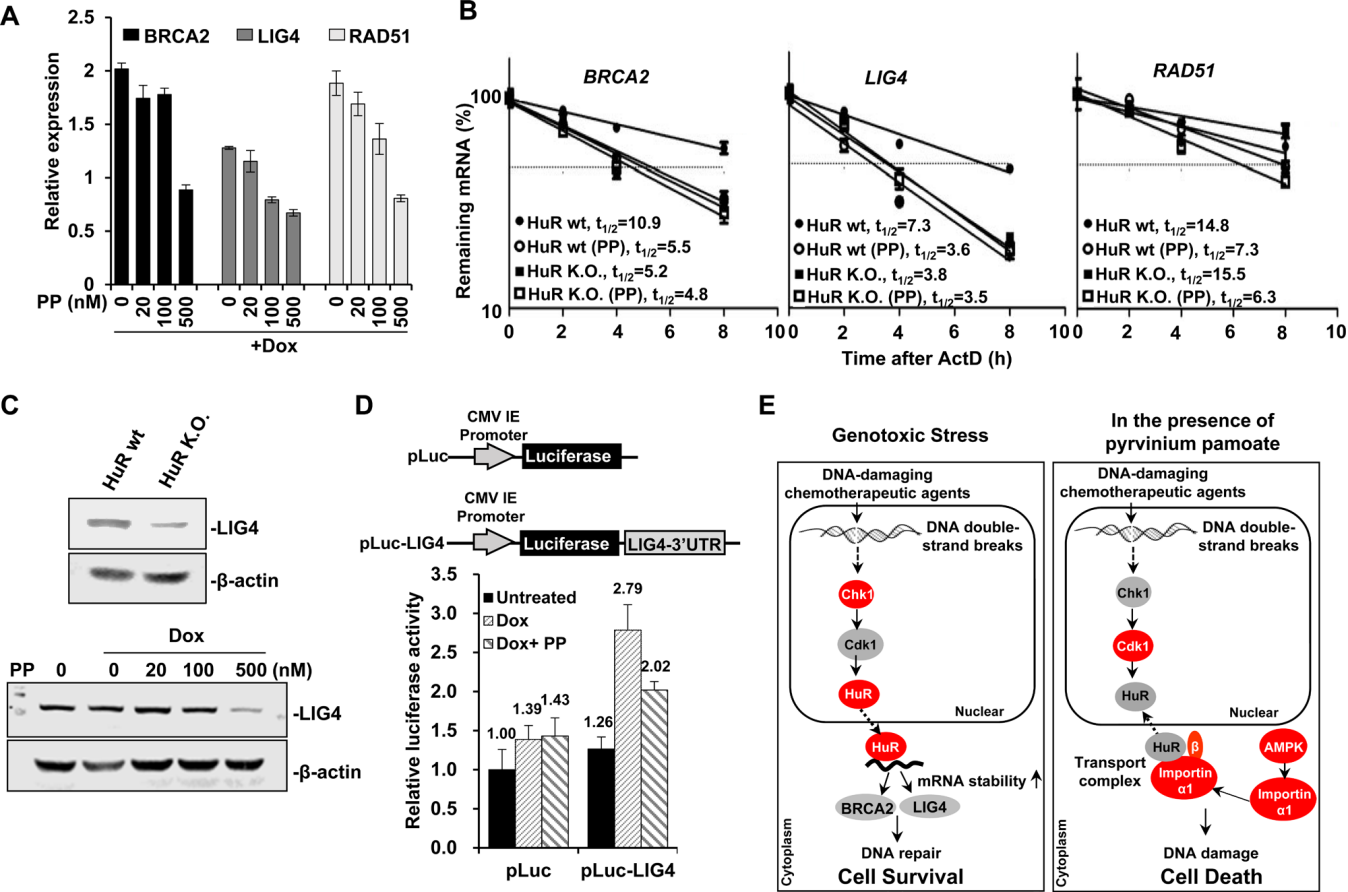
Pyrvinium pamoate suppresses HuR-mediated stabilization of DNA repair genes (**A**) Pyrvinium pamoate dose-dependently inhibits doxorubicin-induced overexpression of *BRCA2*, *LIG4* and *RAD51* genes. The relative changes in gene expression were expressed using untreated cells at 100%. (**B**) Pyrvinium pamoate decreases mRNA stability of *BRCA2*, *LIG4* and *RAD51* genes. (**C**) Pyrvinium pamoate decreases LIG4 protein level in a dose-dependent manner. Upper, LIG4 expression; lower, decreased LIG4 expression by pyrvinium pamoate. (**D**) Pyrvinium pamoate inhibits doxorubicin-induced HuR binding to LIG4 3′UTR. 5637 cells were transiently transfected with either empty vector pLuc reporter plasmid or a 3′UTR reporter construct. *Dots or Columns*, mean of four independent replicates; *bars*, standard deviation. Dox, doxorubicin; PP, pyrvinium pamoate; wt, wild-type; K.O., knockout. (**E**) Schematic diagram of pyrvinium pamoate in potentiating chemotherapeutic efficacy. Grey proteins refer to inactive state and red ones refer to active state.

Two repair systems, HR and NHEJ occur in cells suffering DSBs. We asked if pyrvinium pamoate could downregulate the expression profile of DNA damage repair factors. A panel of differentially expressed genes (enzymes and their regulatory proteins) necessary for HR or NHEJ was detected. The expression of most genes encoding DNA repair factors tended to increase under genotoxic (doxorubicin) stress (Supplementary Table S1), which was consistent with previous studies [31, 32]. However, the expression of these up-regulated ones were decreased once pyrvinium pamoate was added, among which several (breast cancer susceptibility gene (BRCA2), DNA ligase IV (LIG4) as well as recombinase RAD51) exhibited significantly decreased *P*-values as compared with doxorubicin alone group (Supplementary Table S1). We further confirmed the dose-dependent effect of pyrvinium pamoate-mediated downregulation of BRCA2, LIG4 and RAD51 under genotoxic stress (Figure 6A).

HuR typically modulates its target gene expression by affecting mRNA stability [11]. We hypothesized that pyrvinium pamoate might regulate several genes involved in DNA repair through affecting their mRNA stability. As shown in Figure 6B, pyrvinium pamoate treatment led to a doubled rate of mRNA degradation of BRCA2, LIG4 and RAD51, three critical genes in DNA repair. When HuR was absent, BRCA2 and LIG4 mRNAs degraded at an much accelerated rate (10.9 h *vs.* 5.2 h, 7.3 h *vs.* 3.8 h), but not RAD51 (14.8 h *vs.* 15.5 h). To further exclude other possible mechanisms behind pyrvinium pamoate-triggered accelerated degradation of these three mRNAs, we also treated HuR-null cells with pyrvinium pamoate, and found that pyrvinium pamoate did not result in degradation of BRCA2 and LIG4 in HuR-null cells to a great extent as compared with wild-type counterparts or untreated HuR-null cells. However, pyrvinium pamoate still led to degradation of RAD51 mRNA even in the absence of HuR (15.5 h *vs.*6.3 h). These results support the notion that pyrvinium pamoate promoted degradation of BRCA2 and LIG4 mRNAs in a HuR-dependent manner, whereas it possibly affected RAD51 mRNA stability in a HuR-independent manner.

Considering that NHEJ is the major pathway of DSB repair [33] and LIG4 is the key factor in this process, we next studied LIG4 in detail. Our results showed that HuR deletion led to a decrease of LIG4 (Figure 6C, upper), indicating that HuR might positively regulate LIG4. Additionally, pyrvinium pamoate at a relatively higher concentration diminished LIG4 protein level in the presence of doxorubicin (Figure 6C, lower). These results indicated that pyrvinium pamoate suppressed LIG4 expression in a HuR-dependent manner, and LIG4 might be a novel target of HuR. To determine the molecular interaction between HuR and LIG4 mRNA, we cloned the whole sequence of the LIG4 3′UTR into a luciferase construct (Figure 6D). Our results showed that cells harboring a LIG4 3′UTR-containing vector responded to doxorubicin and exhibited a two-fold higher luciferase activity when compared with control cells that lacked the 3′UTR. However, the elevated luciferase activity remarkably decreased once pyrvinium pamoate was added, suggesting that HuR and its downstream LIG4 could be regulated by a HuR inhibitor, such as pyrvinium pamoate.

To elucidate the potential benefit of pyrvinium pamoate-combined strategy in UCB, we detected the expression of several clinical prognostic biomarkers (PIK3CA, AURKA, PFKFB4, and HRAS) and responsive biomarkers (Mre11, BRCA1, MDR1) in UCBPDX0615 xenografts. We found that pyrvinium pamoate combined with cisplatin inhibited expression of all the four prognostic biomarkers compared to the vehicle control groups (Supplementary Figure S6). Additionally, the combined regimen led to a significant decrease of the three responsive biomarkers compared to cisplatin-treated group (*P* < 0.01), indicating an improvement of response to cisplatin therapy by addition of pyrvinium pamoate (Supplementary Figure S6). In this study, we clearly elucidated a paradigm whereby systemic modulation of cytoplasmic abundance of HuR is sufficient to sensitize cancer cells to chemotherapy (Figure 6E).

## DISCUSSION

The RNA binding protein HuR alters its subcellular distribution in response to genotoxic stresses (i.e., radiotherapy and chemotherapy). HuR cytoplasmic accumulation is correlated to poor prognosis of bladder cancer patients [23, 24]. However, no HuR selective inhibitors are available for clinical use until now, leaving it hard to elucidate the impact of HuR on current therapy. In this study, we identified an FDA-approved drug pyrvinium pamoate as a novel HuR inhibitor. We found that pyrvinium pamoate inhibited cytoplasmic accumulation of HuR by a dual mechanism involving the AMPK/importin α1 cascade and the Chk1/Cdk1 pathway. The synergy of pyrvinium pamoate and first-line chemotherapies led to elevated double-strand breaks and blocked the DNA damage response, in part, by targeting HuR.

Given the critical role of HuR in the stabilization of various mRNAs of survival cargo, HuR-interfering strategy should have potential in cancer therapy. Three compounds that inhibit the binding of HuR and its downstream mRNAs were first described in 2007 [15]; however, these compounds exhibited unexpected off-target effects [34, 35]. Recently, a cluster of compounds that disrupted HuR-ARE interaction were identified by a fluorescence polarization assay [36]. Unfortunately, limited efficacy in cells hinders their further preclinical evaluation. A possible explanation for this is that HuR modifications are hard to simulate in a fluorescence polarization assay. In this study, we set up a cell-based and ARE-based luciferase screening assay that mimics the stress response in cancer cells, and further characterized pyrvinium pamoate as an effective HuR inhibitor. The pivotal role of HuR in pyrvinium pamoate-mediated function was evidenced by selective toxicity of pyrvinium pamoate or a combined therapy in a pair of isogenic cells (Figure 1E and 2B).

Modulation of HuR subcellular distribution is a complicated process [26, 37–39]. In this study, we found that importin α1 participated in HuR nuclear import by pyrvinium pamoate, and this was partially dependent on activation of the AMPK/importin α1 cascade (Figure 3). Previous studies revealed that the AMPK activator AICAR decreased cytoplasmic HuR accumulation under UVC stimulation [26]. However, AMPK activation alone seems insufficient to reverse genotoxic agent-triggered HuR cytoplasmic accumulation (Figure 3C). This may be attributed to different modifications on HuR under different stimuli. UVC exposure activated p38, which leads to phosphorylated HuR at T118 and promotes HuR cytoplasmic translocation [40]. In contrast, genotoxic agents seldom activate p38. Under DNA-damaging conditions, the Chk1/Cdk1 signaling pathway is thought to be indispensable for HuR modification and nuclear import [29, 41], as pS202 HuR forms a stable complex with 14-3–3θ [28]. Inhibition of Cdk1 by activated Chk1 caused a decrease in pS202 HuR, while agents, such as pyrvinium pamoate, could reverse this process through inhibiting Chk1 (Figure 4).

HuR regulates nearly 4% of transcript encoding proteins implicated in cell cycle, cell survival, division, migration and angiogenesis [42]. However, whether HuR is implicated in DNA damage response has not been detailedly defined. We found that the expression of many factors participated in homologous recombination and non-homologous end joining was markedly increased by doxorubicin, whereas addition of pyrvinium pamoate significantly decreased their expression (Supplementary Table S1). Among these HuR post-transcriptionally regulated mRNAs, LIG4 was the most striking one due to its potent anti-tumor effect [43]. Our half-life analysis and luciferase assay proved that HuR-recognized motifs exist in the 3′UTR of LIG4 mRNA, unveiling a novel mechanism of acute non-homologous end joining activation under genotoxic stress. Because HuR activation during DNA damage response is also correlated to cell cycle regulator expression [19–22], interfering with HuR is a promising strategy to combat cancer.

We reported the strong synergy of pyrvinium pamoate and first-line chemotherapy in preclinical bladder cancer mouse models. When combined with the chemotherapy drug cisplatin, pyrvinium pamoate significantly contributed to a durable cytostatic tumor growth response (Figure 2D). The UCBPDX0826 was derived from primary bladder tumors, while UCBPDX0615 was derived from tumors undergoing neo-adjuvant chemotherapy. Consistent with parent tumor characteristics, the UCBPDX0826 xenografts demonstrated a better response to cisplatin monotherapy, whereas UCBPDX0615 showed poor response to cisplatin. Most strikingly, pyrvinium pamoate-additive regimen dramatically sensitized UCBPDX0615 to cisplatin and efficiently enhanced tumor regression. These results support the notion that a combined regimen of pyrvinium pamoate and chemotherapy is highly effective, particularly to treat resistant or unresponsive tumors. Notably, the mice bearing primary bladder tumor xenografts suffered no decrease of body weight or other obvious side effects at tested dosages. The safety of our HuR interfering strategy was also supported by other studies [35, 44], suggesting that HuR is a safe target. A series of bladder cancer clinical biomarkers were inhibited by the addition of pyrvinium pamoate (Supplementary Figure 6), suggesting a potential clinical benefit of pyrvinium pamoate-addition regimen.

## MATERIALS AND METHODS

Detailed protocols are provided in Supplementary Materials and Methods.

### Cell culture

Bladder cancer cell lines 5637 and T24 were purchased from American Type Culture Collection (Manassas, VA) and cultured in RPMI-1640 (Gibco, Grand Island, NY) and McCoy's 5A Medium (Sigma, St Louis, MO), respectively. The 293T cell line was cultured in Dulbecco's Modified Eagle Medium (DMEM; Gibco). All the cells were grown in the media containing 10% fetal bovine serum (Gibco) and 1% penicillin/streptomycin (Invitrogen, Carlsbad, CA) at 37°C under a humidified 95:5 (%; v/v) mixture of air and CO_2._ Cells were authenticated by short tandem repeat analysis before use.

### Cytolasmic and whole-cell extracts

#### Cytoplasmic extracts

Bladder cancer cells were collected using trypsin digestion and incubated on ice for 15 min in cytoplasmic lysis buffer (10 mmol/L HEPES-NaOH, 10 mmol/L KCl, 1.5 mmol/L MgCl_2_ and 0.5 mmol/L beta-mercaptoethanol) supplemented with protease and phosphatase inhibitors. Lysates were then incubated with 10% NP-40 and centrifuged at 16000 g for 15 min. The supernatant was collected.

#### Whole-cell extracts

Bladder cancer cells were treated with drugs for indicated time intervals and then lysed in cold radioimmunoprecipitation assay buffer (RIPA buffer; 20 mmol/L Tris, 2 mmol/L ethylenediaminetetraacetic acid, 1% Triton X-100, 1% sodium deoxycholic acid and 0.1% sodium dodecyl sulfate) containing a proteinase inhibitor cocktail. Lysates were centrifuged at 12,000 rpm for 20 min at 4°C and the supernatant was collected.

### Drug sensitivity and growth assay

#### Drug sensitivity assay

The effect of drugs on cell viability was measured using CellTiter 96^®^ AQueous One Solution from Promega (Madison, WI) as previously described [45]. The IC_50_ values were calculated by Prism 5 (GraphPad Software, La Jolla, CA). The drug combinations were performed with fixed drug ratios (i.e., the IC_50_ ratio), and the combination index (CI) as described by Chou-Talalay [46] was generated using CalcuSyn software (Version 2; Biosoft). Combinations with CI_s_ < 1 were considered to be synergistic.

#### Anchorage independence growth assay

5637 cells suspended in 0.35% low melting point agarose/growth medium were planted onto 6-well plates (500 cells/well) with a 0.6% agarose underlay. Growth medium containing different drugs or drug pairs was then added on the top of agarose. Medium was changed every three days for total 15 days.

#### Luciferase assay

Luciferase assays were performed as previously described [22]. Briefly, PCR products of the AU-rich element or full-length LIG4 3′UTR were purified and cloned into modified pGL3-basic vector. 5637 cells were transfected with these plasmids, followed by further drug treatments at 48 h post-transfection. 5637 cells transfected with modified pGL3 that contains firefly luciferase but lacks the 3′UTR were used as the control.

#### Immunofluorescence and Immunohistochemistry

The translocation of HuR was examined by immunofluorescence assays. See Supplementary Materials and Methods for detail protocols.

#### Alkaline comet assay

The 5637 cells were treated with indicated drugs for 48 h and collected. The rate of DNA damage was measured using Comet assay kit (Trevigen, Gaithersburg, MD) following the manufacturer's instructions. Imaged were taken by a fluorescence microscope (Leica, Bensheim, Germany).

#### PCR and quantitative real-time PCR

The total RNA from treated cells was collected using RNAiso plus (TaKaRa Biotechnology, Dalian, China). RNA was then converted to cDNA using the PrimeScript RT reagent kit (TaKaRa). Quantitative real-time PCR was performed according to the manufacturer's instructions of the SYBR^®^ Premix Ex Taq kit (TaKaRa) using the 96-well Thermal iCycler (Biorad, Hercules, CA). Sequences of primers are listed in Supplementary Table S2 and Supplementary Table S3.

#### Analysis of mRNA stability

mRNA stability was determined by measuring the amount of mRNA at various time points after actinomycin D was added [22]. 5637 cells were exposed to pyrvinium pamoate (100 nmol/L) for 24 h, followed by treatment with 5 μmol/L of actinomycin D for indicated time points (0, 2, 4, 8 h). The threshold cycle number for target mRNA was normalized to that of β-actin, and the values were converted to a linear scale. All assays were performed at least three times from independent RNA preparations.

#### Statistical analysis

The data are presented as the mean ± standard deviation. Statistical tests were performed using Microsoft Excel and GraphPad Prism Software version 5.0 (GraphPad Software Inc., San Diego, CA). For two group comparisons, a two-tailed unpaired *t*-test was used. For multiple group comparisons, a One-way ANOVA was used. *P* < 0.05 was considered as significant.

## Supplementary Materials Tables and Figures


