# Supplementary material for: Inhibiting cytoplasmic accumulation of HuR synergizes genotoxic agents in urothelial carcinoma of the bladder
Source: Oncotarget. 2016 Jun 9;7(29):45249–62. doi: 10.18632/oncotarget.9932 (PMC5216720; doi:10.18632/oncotarget.9932)
Supplement: Supplementary file 1 [file oncotarget-07-45249-s001.pdf]

Inhibiting cytoplasmic accumulation of HuR synergizes genotoxic agents in urothelial carcinoma of the bladder

Supplementary Materials

MATERIALS AND METHODS

Reagents

Pyrvinium pamoate and actinomycin D were purchased from Sigma (St. Louis, MO). Chemotherapeutic agents (doxorubicin, cisplatin, oxaliplatin, vincristine and gemcitabine) were bought from Selleck (Houston, TX). Unless otherwise specified, the half maximal inhibitory concentrations (IC_{50}) of chemotherapeutic agents and pyrvinium pamoate were used. Sodium azide, 5-amino-imidazole-4-carboxamide riboside (AICAR, Selleck), compound C (Selleck) and AZD5438 (MCE, Lake Placid, NY) were added in to cell culture medium at a final concentration of their half maximal effective concentrations or IC_{50s} on their target kinases. Bacto® agar was obtained from BD Biosciences (San Jose, CA). ANTI-FLAG® M2 affinity gel was purchased from Sigma.

Antibodies

Antibody against HuR was purchased from Santa Cruz Biotechnology (Santa Cruz, CA). Antibodies against AMPK, γH2AX, phospho-Chk1 (S345) and β-actin were purchased from Cell Signaling Technology (Danvers, MA). Antibodies against phospho-AMPKα (T172), Chk1, Cdc25C, myc-tag, flag-tag M2, histone H3 and α-tublin were purchased from Abcam (Cambridge, MA). Antibody against phospho-Cdk1 (T161, Y15) was purchased from Abclonal technology (Cambridge, MA). Antibody against Cdk1 was purchased from Sigma.

Plasmids construction

Human HuR cDNA (encoding full-length HuR) and KPNA2 cDNA (encoding importin α1 protein) were amplified by the polymerase chain reaction (PCR) from the total RNA of 5637 bladder cancer cells and cloned into the mammalian expression vector pcDNA3.0-5′-flag and pcDNA3.1/myc-His (Invitrogen), respectively. ARE and 3′ UTR of LIG4 were cloned into the 3′UTR of the luciferase gene in pGL3-basic (Promega, Madison, WI). For gene overexpression, HuR (wild-type/S202A/S202D) and importin α1 (wild-type/K22R/S105A) were cloned into pcDNA3.1 vector (Invitrogen). Plasmids were all transfected using Lipofectamine 2000 (Invitrogen).

HuR knockout cell lines

The SpCas9 targeting vector lentiCRISPR v2 was obtained from Addgene (Cambridge, MA). HuR sgRNA (ACCACATGGCCGAAGACTGC) was designed using the Optimized CRISPR Design Tool (Zhang lab, MIT). Lentiviruses were produced by co-transfection of lentiviral backbone constructs (lentiCRISPR v2) and packaging vectors (pMD2.G and pSPAX2; Addgene) into 293T cells. Supernatant was collected after 48 h post-transfection and consequently added to bladder cancer cells in a 6-well plate. Infected cells were selected with puromycin (Sigma). Single cells with HuR frame-shift mutations were selected.

Primary bladder tumor xenograft mouse models

The mice used in the present study were purchased from National Rodent Laboratory Animal Resources (Shanghai, China). Animals were caged in groups of 5 in a laminar airflow cabinet under specific pathogen-free conditions, fed with sterilized food and water and kept on a 12-hour light/dark cycle. All treatments were administered according to the guidelines of Institution Animal Care and Use Committee and all the protocols were approved by East China Normal University.

For establishment of UCB patient-derived xenograft mouse model, fresh tumor samples from 23 bladder cancer patients were obtained from the Shanghai Changhai Hospital (Shanghai, China). Prior written informed consent was obtained from all patients, and the study protocol was approved by the local hospital ethics committee. In brief, surgically removed tumor tissues were cut into fragments (approximately 2 mm^3) and implanted subcutaneously into the flanks of male nude mice. When tumor volume reached approximately 1000-1500 mm^3, primary xenografts (designated as P0) at exponential growth phase were removed by serial passage to other immunodeficient mice (designated as

P1 to P6). Histological morphology of two primary UCB tumor xenograft models was characterized by hematoxylin and eosin staining, and recurrent UCB mutations were sequenced as well. In this study, UCBPDX0615 model (P4; pT1 stage; TP53, FGFR3, PIK3A mutation) and UCBPDX0826 model (P3; pT2b stage; TP53, FGFR3, HRAS mutation) were applied to drug treatments.

Primary tumor-bearing mice with an average tumor volume reaching approximately 200 mm^3 were randomly divided into the following groups (n = 8–12 each group): vehicle control (phosphate-buffered saline; i.p.; daily); pyrvinium pamoate (0.8 mg/kg; i.p.; daily); cisplatin (4 mg/kg; i.p.; weekly); or a combination of pyrvinium pamoate and cisplatin at the same dosages as single treatments. All groups were continuously treated for 4 weeks, and the body weight was measured every other day. Tumor size was evaluated by caliper measurements, and the approximate volume of the tumor mass was calculated using the following formula: $V = (L \times [W]^2) \times 0.52$; where L is the longest diameter of the tumor; and W is the shortest diameter of the tumor. At the end of the experiment, solid tumors were removed, weighed and processed for immunohistochemistry and immunofluorescence.

Immunoprecipitation assay and Western blotting assay

For immunoprecipitation assay, 5637 cells were co-transfected with flag-HuR and myc-importin α1. Meanwhile, cells co-transfected with pcDNA3.0-5′-flag and myc-importin α1 vectors served as negative controls. Two days after transfection, cells were exposed to indicated treatments followed by lysed with EBC buffer (20 mmol/L Tris-HCl, 125 mmol/L NaCl, 2 mmol/L EDTA, and 0.5% NP-40) supplemented with protease inhibitors. Cell lysates were consequently incubated with protein A-conjugated sepharose beads and the anti-flag M2 agarose beads at 4°C with rotation. Beads were boiled with SDS sample buffer and subjected to Western blotting assays.

For Western blotting assays, the whole-cell extracts were prepared in RIPA buffer. Approximately 40–50 μg of cellular protein from each treatment was applied to 12% SDS-polyacrylamide gels and transferred onto nitrocellulose membranes (Millipore, Billerica, MA). Membranes were incubated overnight with primary antibodies followed by incubation with fluorescent secondary antibodies. After several washes, the signals were detected by the Li-Cor Odyssey Infrared system (LI-COR Biosciences, Lincoln, NE).

Immunofluorescence and Immunohistochemistry

Treated cells were plated onto gelatin-coated coverslips and fixed with 3.7% paraformaldehyde for 15 min, permeabilized with 0.1% Triton X-100 for 15 min, and then blocked with 1% bovine serum albumin for 1 h. Cells were then subjected to specific primary and secondary antibodies. Phalloidin and 4, 6-diamidino-2-phenylindole (Thermo Scientific, Grand Island, NY) were further used to stain cytoskeleton and nuclei. The images were acquired using a laser confocal microscope (Zeiss Axiovert 200M LSM510).

The expression of HuR and γH2AX in treated tumors was detected by immunohistochemistry and immunofluorescence, respectively. Primary tumors were fixed, embedded in paraffin and were sectioned (4-μm). Randomly selected slides were stained with HuR and γH2AX antibodies following to standard protocols. Images were obtained with Leica microscope (Leica, DM4000b).

Supplementary Table S1: Pyrvinium pamoate downregulates DNA repair genes under genotoxic stress (Related to Figure 6A)

Symbol	*P* value		Gap		Gene name
	Dox *vs.* control	Dox + PP *vs.* Dox	Dox *vs.* control	Dox + PP *vs.* control	
LIG4	0.0004	0.0003	1.43	0.91	DNA Ligase IV
RAD51	0.0016	0.0016	1.42	1.17	RAD51 recombinase
BRCA2	1.04E-05	0.0070	0.86	0.59	breast cancer type 2 susceptibility protein
DNA-PKcE	0.0027	0.0079	1.89	1.34	DNA-dependent protein kinase, catalytic subunit
Chk1	0.0001	0.0117	1.92	1.53	checkpoint kinase 1
PARP1	1.20E-05	0.0163	1.82	2.26	poly (ADP-ribose) polymerase 1
MRE11	0.0628	0.0344	1.27	1.54	MRE11 homolog A, double strand break repair nuclease
hSSB1	0.0008	0.0477	1.85	1.53	human single-stranded binding protein 1
53BP1	0.1137	0.0566	1.39	0.9	p53-binding protein 1
XRCC6	0.5628	0.1531	1.08	0.82	X-ray repair cross-complementing protein 6
XRCC4	0.2964	0.1686	1.02	0.96	X-ray repair cross-complementing protein 4
ERCC2	0.6143	0.1712	1.07	0.8	excision repair cross-complementation group 2
BRCA1	0.331	0.2133	2.01	1.96	breast cancer type 1 susceptibility protein
RAD50	0.0046	0.2179	2.03	1.73	RAD50 homolog, double strand break repair protein
NBS1	0.0077	0.4596	0.78	0.83	nibrin
XLF	0.0426	0.5189	1.52	1.67	XRCC4-like factor
RPA	0.0199	0.8399	1.27	1.25	replication protein A
ATM	0.0029	0.9797	1.5	1.49	ataxia telangiectasia mutated

NOTE: *P* values were calculated by *t*-tests. Gap is the difference of median derived from log-transformed relative expression to the untreated control group. Dox, doxorubicin; PP, pyrvinium pamoate.

Supplementary Table S2: Sequences of primers for PCR amplification

Genes	Forward (5′-3′)	Reverse (5′-3′)
LIG4 3′UTR	CCGGAATTCCAGACTCATTGCAGCAGGTG	TCCCCCCGGGTCACATACATTTGTTCCACGGT
HuR	CCCTCGAGCATGTCTAATGGTTATGA	CTCGAGTTTGTGGGACTTGTTGGTTTTGAAG
TNPO2	GAATTCTTATGGACTGGCAGCCAGACG	CTCGAGCTAGACCCCATAGAAAGCCG
KPNA2	TATAAGCTGGTGGTGGTGGG	TGATGGCAAACACACACAGG
KPNA2 S105D	CCAGGAAACTACTTGACAGAGAAAAACAGC	GCTGTTTTTCTCTGTCAAGTAGTTTCCTGG
KPNA2 K22Q	CAAGAACAAGGGACAAGACAGTACAGAAAT	ATTTCTGTACTGTCTTGTCCCTTGTTCTTG
CDK1(EcoRI/ Xhol)	GAATTCCCATGGAAGATTATACC	CTCGAGCTACATCTTCTTAAT
HuR(Xhol/ EcoRI)	CTCGAGATGTCTAATGGTTATG	GAATTCTTCTTTGTGGGACTTGT
HuR S202D	CGCAGCTGTACCACAACCCAGCGCGACGGT	ACCGTCGCGCTGGGTTGTGGTACAGCTGCG
HuR S202A	CGCAGCTGTACCACGCGCCAGCGCGACGGT	ACCGTCGCGCTGGCGCGTGGTACAGCTGCG
HuR mPAM	GGCCGAAGACTGCAGAGGTGACATCGGGAG	CTCCCGATGTCACCTCTGCAGTCTTCGGCC

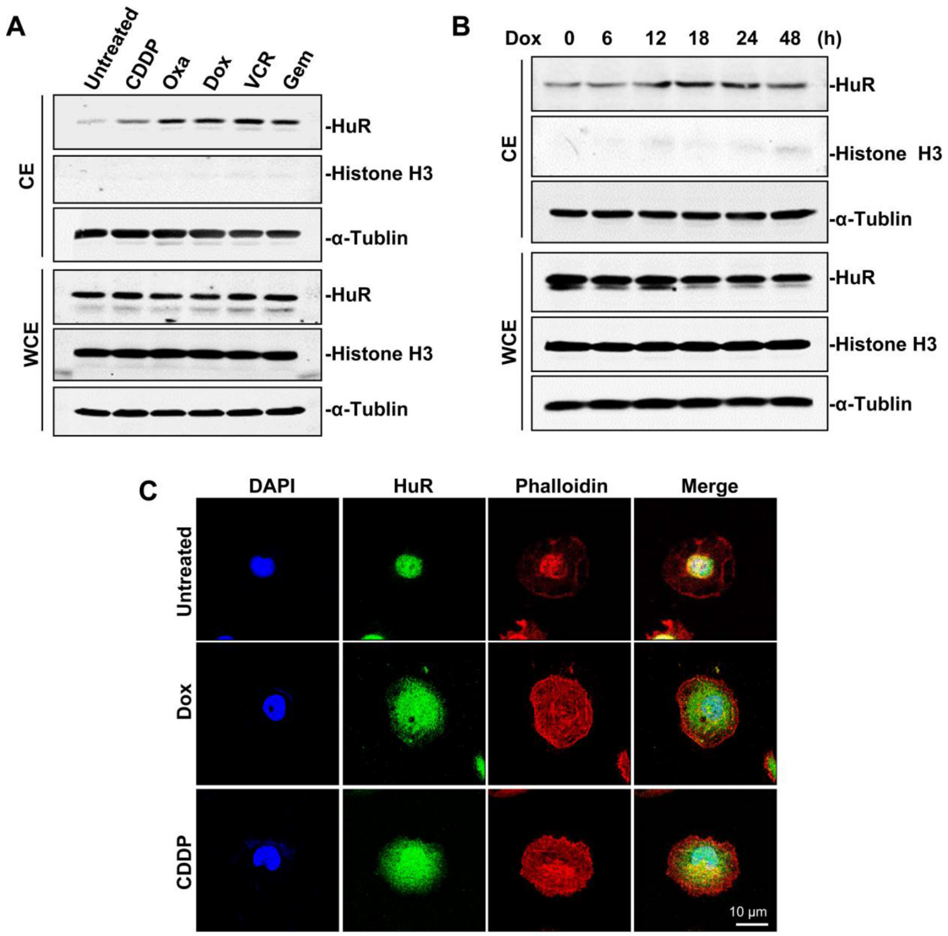

Supplementary Figure S1: HuR well-responds to the treatments of genotoxic agents (Related to Figure 1). (**A**) Increased cytoplasmic accumulation of HuR after different treatments. 5637 cells were respectively treated with 6.8 μmol/L of cisplatin, 1.8 μmol/L of oxaliplatin, 60 nmol/L of doxorubicin, 9 nmol/L of vincristine and 2.8 μmol/L of gemcitabine for 24 h. Cell lysates were collected and then subjected to Western blotting assays. (**B**) HuR cytoplasmic translocation is time-dependently increased by doxorubicin. (**C**) Increased HuR cytoplasmic translocation triggered by cisplatin and doxorubicin by immunofluorescence. CE, cytoplasmic extracts; WCE, whole-cell extracts; CDDP, cisplatin; Oxa, oxaliplatin; Dox, doxorubicin; VCR, vincristine; Gem, gemcitabine.

Supplementary Table S3: Sequences of primers for quantitative real-time PCR analysis

	Genes	Forward (5′-3′)	Reverse (5′-3′)
**Prognosis markers**	PIK3CA	GGAGCCCAAGAATGCACAAA	TTTGTTGTCCAGCCACCATG
	AUROKA	GAAGCAATTGCAGGCAACCA	GAGGGCGACCAATTTCAAAG
	PFKFB4	TGAGGTCATAGCTGCCAACA	GGGTCACGTGGATGTTCATG
	HRAS	TATAAGCTGGTGGTGGTGGG	TGATGGCAAACACACACAGG
**Responstive markers**	Mre11	TGGGTAATTCTCACCAGCCA	GCACATTCTTCTCTGCGGTT
	BRCA1	TTCAAGGTTTCAAAGCGCCA	CTGACCAACCACAGGAAAGC
	MDR1	TTCTACGGGAAATCATTGGTGTG	CTGTCTGCATTGTGACAAGTTTG
**Target confirm**	DNA Ligase IV	GCAGAGATCGTACCCAGTGA	GATGCGAGCTTACCAGATGC
	BRCA2	CTGTCTCAGCCCAGATGACT	TTTGGTGCCACAACTCCTTG
**The DNA damage response -related enzymes and proteins**	hSSB1	GACAGCAACCCTTCAGCTTC	TATCTCTTGCTGCTCCTCCG
	Mre11	TGGGTAATTCTCACCAGCCA	GCACATTCTTCTCTGCGGTT
	Rad50	GCGTGCGGAGTTTTGGAATA	AGCAACCTTGGGATCGTGTA
	NBS1	AACACAACCTGCTACACCCT	GGCCACATCATCCATTTCCC
	ATM	AGTACCCCTTGCCAATGGAA	TCCTTGAGCATCCCTTGTGT
	RPA	ATCCGTACCTGGAGCAACTC	ATCTTCAGGGTGCCTTTCGA
	Rad51	ACCGCCCTTTACAGAACAGA	TGGGATCAGCAGCAAACATC
	Chk1	TCAGACTTTGGCTTGGCAAC	CAGCGAGCATTGCAGTAAGT
	PARP1	TGGAACATCAAGGACGAGCT	CATCGCTCTTGAAGACCAGC
	DNA-PKcs	CTGTGTGAACTGGTTGCGAA	TCATTCCCTCCACACGACAA
	Ku70/80	CAGTGAAAACCAAGACCCGG	CAACGGCTTGAAACCCATGA
	XRCC4	ATGACTGCTGACCGAGATCC	TCCTGAGGAGCCATTTTAGGT
	XLF/cer	CTCCTGTGATTGTGTGGCAG	TAATGCCAGACTCATGCCCA
	53BP1	TGGAAGCTCAGGGAAAGGAG	ACCATCCTCCTCACACACTG
**HuR downstream mRNAs**	P53	AGGTTGGCTCTGACTGTACC	TCTTCTTTGGCTGGGGAGAG
	P21	CGACTGTGATGCGCTAATGG	AGGCACAAGGGTACAAGACA
	COX-2	CCCAGGGCTCAAACATGATG	GCAAACCGTAGATGCTCAGG
	Bcl-2	GCCTTCTTTGAGTTCGGTGG	CTTCAGAGACAGCCAGGAGA
	PTMA	GAAAATGGAAGAGACGCCCC	TCGTCGGTCTTCTGCTTCTT

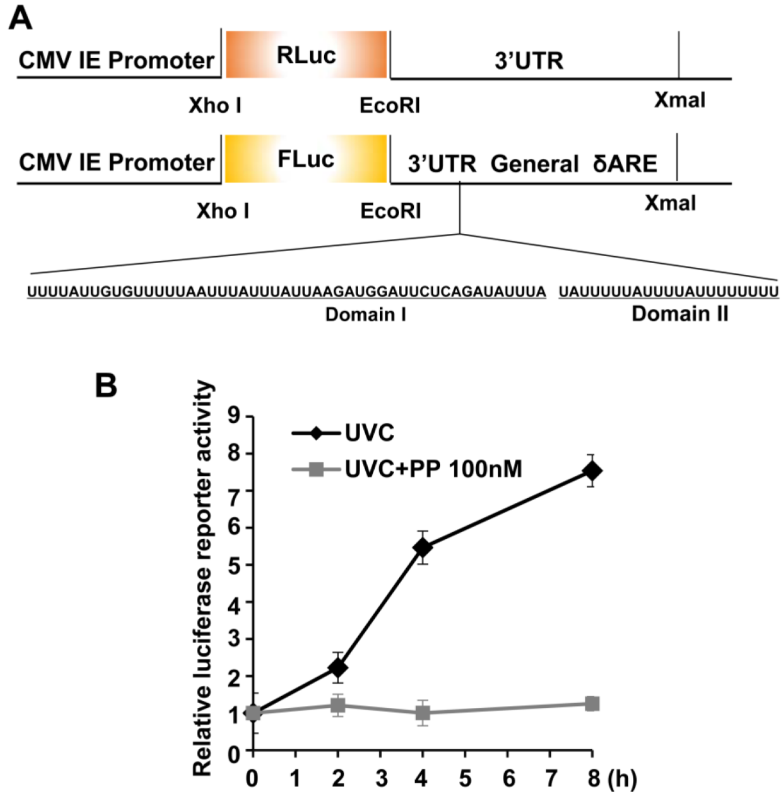

Supplementary Figure S2: Pyrvinium pamoate decreases the FLuc/RLuc ratio (Related to Figure 1B). (**A**) Schematic diagram of luciferase screening for HuR modulators. The ratio of FLuc value to RLuc value was considered as the readout. (**B**) Pyrvinium pamoate inhibits UVC-mediated increases of the FLuc/RLuc ratio in a time-dependent manner. 5637 cells transfected with equivalent amount of pFLuc and pRLuc were treated with pyrvinium pamoate (100 nmol/L) after exposure to UVC irradiation (20 J/m^2). *Dot,* mean; *bars,* standard deviation. FLuc, Firefly luciferase; RLuc, Renilla luciferase; PP, pyrvinium pamoate.

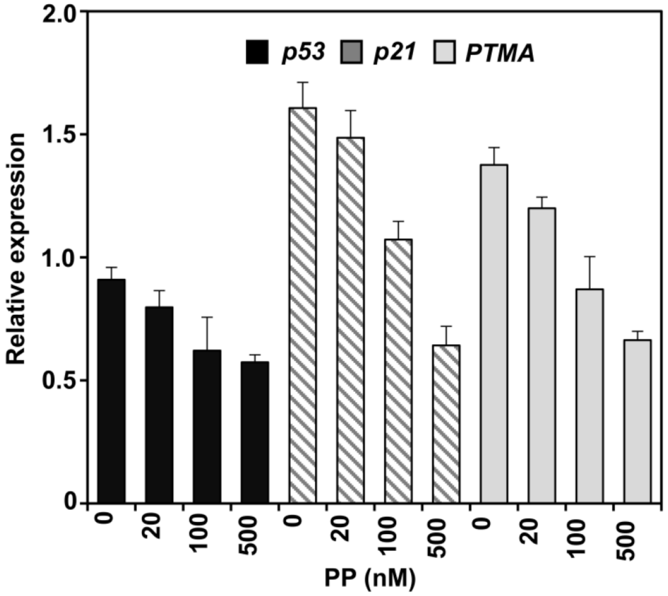

Supplementary Figure S3: Pyrvinium pamoate decreases transcription of endogenous HuR target mRNAs (Related to Figure 1B). 5637 cells were exposed to UVC (20 J/m^2) and then incubated with indicated concentrations of pyrvinium pamoate for 24 h. Quantitative real-time PCR analysis on *p53*, *p21* and *PTMA* was further conducted. The relative changes in gene expression was expressed using untreated cells at 100%. *Columns*, mean of four independent replicates; *bars*, standard deviation. PP, pyrvinium pamoate.

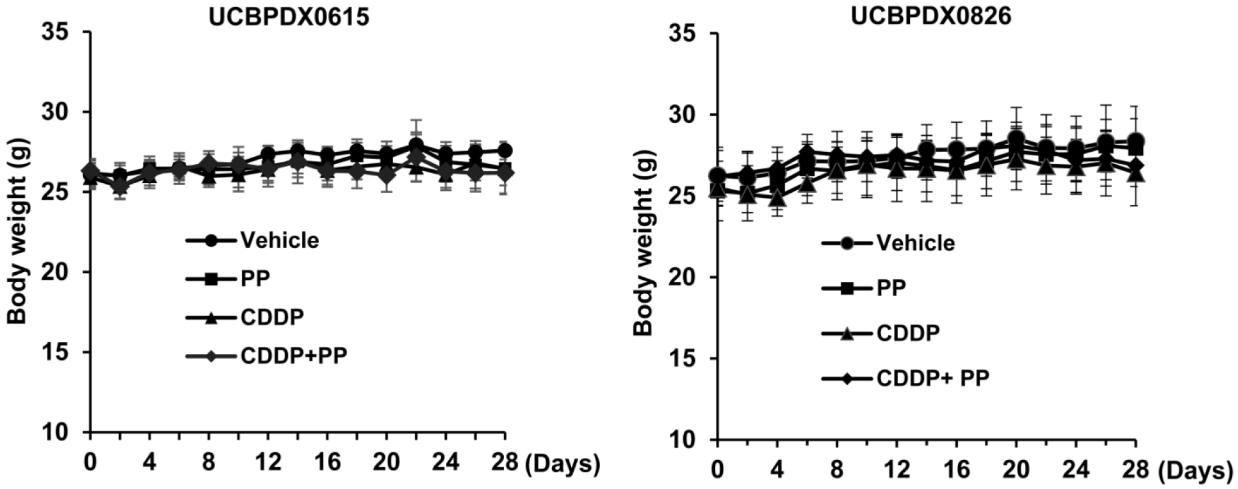

Supplementary Figure S4: Body weight in primary bladder cancer xenograft mice is not significantly affected by treatments (Related to Figure 2D and 2E). Mouse body weight were measured on indicated days (n = 8–12 each group). *Dot,* mean; *bars,* standard deviation. CDDP, cisplatin; PP, pyrvinium pamoate.

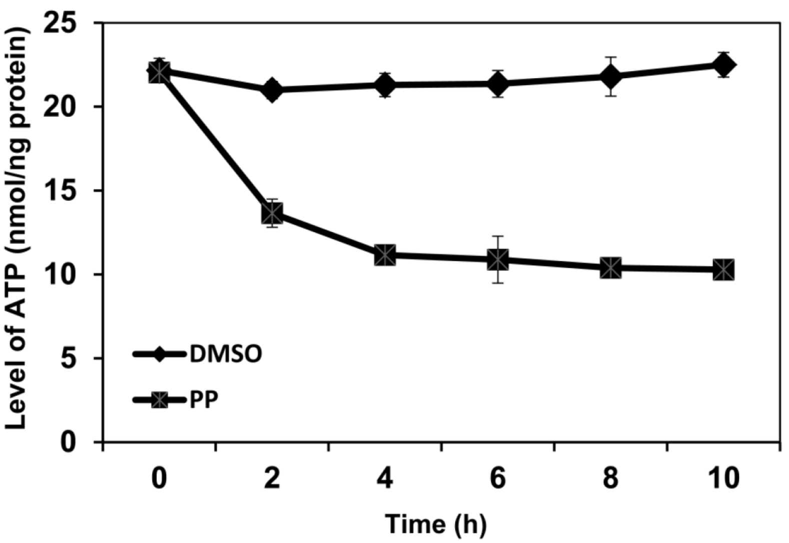

Supplementary Figure S5: Pyrvinium pamoate decreases ATP production (Related to Figure 3A). 5637 cells were treated with 100 nmol/L of pyrviunium pamoate for indicated hours. ATP level was then measured and normalized to cell protein quantity. *Dots*, mean of four independent replicates; *bars*, standard deviation. PP, pyrvinium pamoate.

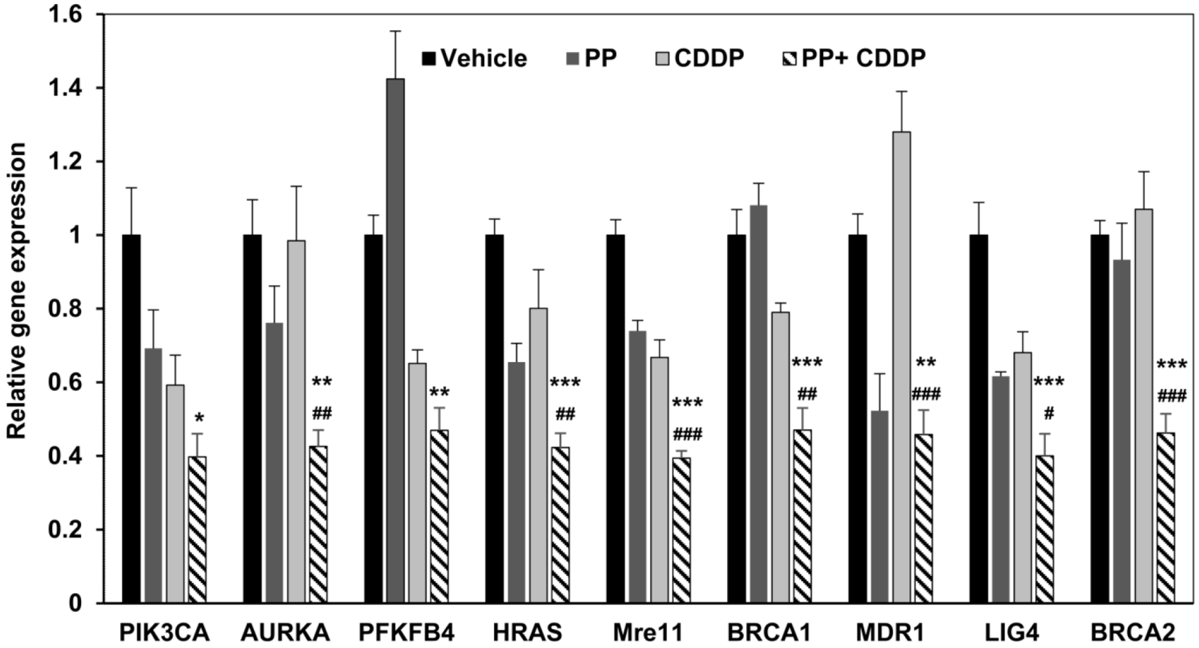

Supplementary Figure S6: Pyrvinium pamoate-additive treatment decreases the expression of prognostic markers of bladder cancer. cDNAs extracted from UCBPDX0615 solid tumors were used for quantitative real-time PCR. Statistical comparisons of gene expressions in combined treatment groups with all other arms were performed by One-way ANOVA analysis. *Columns*, mean of four replicates; *bars*, standard deviation. *indicates significance between combinative group and the vehicle group; $*P < 0.05$; $**P < 0.01$; $***P < 0.001$. #indicates significance between the combined treatment group and the cisplatin alone group; $^{\#}P < 0.05$; $^{\#\#}P < 0.01$; $^{\#\#\#}P < 0.001$. CDDP, cisplatin; PP, pyrvinium pamoate.
